# An Indispensable Role for the MavE Effector of Legionella pneumophila in Lysosomal Evasion

**DOI:** 10.1128/mBio.03458-20

**Published:** 2021-02-09

**Authors:** Bethany Vaughn, Kevin Voth, Christopher T. Price, Snake Jones, Mateja Ozanic, Marina Santic, Miroslaw Cygler, Yousef Abu Kwaik

**Affiliations:** a Department of Microbiology and Immunology, University of Louisville, Louisville, Kentucky, USA; b Department of Biochemistry, University of Saskatchewan, Saskatoon, Saskatchewan, Canada; c Faculty of Medicine, Department of Microbiology and Parasitology, University of Rijeka, Rijeka, Croatia; d Center for Predictive Medicine, College of Medicine, University of Louisville, Louisville, Kentucky, USA; University of Illinois at Chicago

**Keywords:** MavE, lysosomal evasion, trafficking, Legionnaires’, NPxY

## Abstract

Diversion of the Legionella pneumophila-containing vacuole (LCV) from the host endosomal-lysosomal degradation pathway is one of the main virulence features essential for manifestation of Legionnaires’ pneumonia. Many of the ∼350 Dot/Icm-injected effectors identified in L. pneumophila have been shown to interfere with various host pathways and processes, but no L. pneumophila effector has ever been identified to be indispensable for lysosomal evasion. While most single effector mutants of L. pneumophila do not exhibit a defective phenotype within macrophages, we show that the MavE effector is essential for intracellular growth of L. pneumophila in human monocyte-derived macrophages (hMDMs) and amoebae and for intrapulmonary proliferation in mice. The *mavE* null mutant fails to remodel the LCV with endoplasmic reticulum (ER)-derived vesicles and is trafficked to the lysosomes where it is degraded, similar to formalin-killed bacteria. During infection of hMDMs, the MavE effector localizes to the poles of the LCV membrane. The crystal structure of MavE, resolved to 1.8 Å, reveals a C-terminal transmembrane helix, three copies of tyrosine-based sorting motifs, and an NPxY eukaryotic motif, which binds phosphotyrosine-binding domains present on signaling and adaptor eukaryotic proteins. Two point mutations within the NPxY motif result in attenuation of L. pneumophila in both hMDMs and amoeba. The substitution defects of P^78^ and D^64^ are associated with failure of vacuoles harboring the mutant to be remodeled by the ER and results in fusion of the vacuole to the lysosomes leading to bacterial degradation. Therefore, the MavE effector of L. pneumophila is indispensable for phagosome biogenesis and lysosomal evasion.

## Abstract (precis)

Intracellular proliferation of Legionella pneumophila within a vacuole in human alveolar macrophages is essential for manifestation of Legionnaires’ pneumonia. Intravacuolar growth of the pathogen is totally dependent on remodeling the L. pneumophila-containing vacuole (LCV) by the ER and on its evasion of the endosomal-lysosomal degradation pathway.

## INTRODUCTION

Intracellular pathogens that reside in vacuoles within macrophages have evolved mechanisms to evade the endosomal lysosomal degradation (1, 2) and autophagy pathways (3) as well as other innate defense pathways (4, 5). Cytosolic pathogens have also evolved mechanisms for cytosolic detection by the innate defense processes of macrophages (6).

Legionella pneumophila is a Gram-negative environmental bacterium, naturally infecting amoebae in water sources (7). The bacterium evades lysosomal degradation by amoebae and proliferates intracellularly (7). L. pneumophila can become aerosolized allowing for environmental transmission to human hosts. Once inhaled into the lungs, L. pneumophila infects resident alveolar macrophages, causing a severe pneumonia known as Legionnaires’ disease (8–10). Within macrophages, L. pneumophila grows in a membrane-bound vesicle known as the *Legionella*-containing vacuole (LCV) (11, 12). The LCV fuses with endoplasmic reticulum (ER)-derived vesicles and evades lysosomal fusion (13). Proliferation of L. pneumophila within the LCV is dependent on the Dot/Icm type IV secretion system (T4SS) (14, 15), which translocates ∼350 effector proteins into the host cell (12, 16–19). These injected effectors have evolved to manipulate various host cell processes in order to remodel the host cell into a proliferative niche for pathogen proliferation (11, 20). The loss of function of this secretion system causes defective phagosome biogenesis in terms of recruitment of ER-derived vesicles to the LCV and in rapid fusion of the LCV to the lysosomes (11, 14, 21).

The ability of the LCV to be remodeled into an ER-derived vacuole and evade degradation through the endosomal-lysosomal pathway is a key virulence determinant of L. pneumophila (11, 22, 23). However, the mechanism L. pneumophila employs to evade lysosomal fusion is still unclear, but it is regulated at various levels (24). Multiple Dot/Icm translocated effectors directly regulate vesicular trafficking associated with the early secretory system (19, 25–27). Few of the T4SS effector proteins, such as DrrA/SidM, LidA, VipD, and LepB have been shown to be partially required for lysosomal evasion (26, 28, 29). Many of these effectors interact with small host GTPases and/or Rab proteins, which are prominent targets of L. pneumophila effector proteins (30–33). However, to date, no known effector has proven to be indispensable for evasion of the endosomal-lysosomal degradation pathway. The prevalence of functional redundancy among the 350 L. pneumophila effectors suggests that key host pathways are targeted by L. pneumophila (34), and many of these pathways are highly conserved through evolution from unicellular eukaryotes, such as amoeba, to mammals (35, 36). Redundancy of effectors occurs in many different manners, including molecular mimicry, targets, pathways, cellular processes, and system redundancy (37). However, the redundancy of effectors likely represents a tool box for L. pneumophila to replicate within diverse environmental hosts; having specific effectors for certain hosts and some of the amoeba-adapted effectors may have unpredicted paradoxical effects on human macrophages (7, 11, 37–39).

The MavE (*lpg2344*) effector was first identified in a screen aimed at identification of new translocated substrates based on the presence of a glutamate-rich motif found in more than half of L. pneumophila effectors (19). While the amino acid sequence of MavE has no homology to known proteins, a BLAST search revealed MavE is widely distributed throughout the *Legionella* genus, but its role during infection remains unknown (40, 41). Recently, a yeast two-hybrid screen revealed that MavE has a direct interaction with another effector protein, a metaeffector, YlfA/LegC7 (19, 42). YlfA/LegC7 modulates ER vesicle trafficking events (43). YlfA/LegC7 along with two other effectors, YlfB/LegC2 and LegC3, assemble as a complex on the LCV that interacts with ER-derived vesicles to initiate membrane fusion (43–45). Single *ylfA* and *ylfB* mutants and a *ylfA-ylfB* double mutant replicate similar to the wild-type (WT) strain in macrophages (46). However, in single cell assays, the *YlfA-YlfB* double mutant exhibits ∼30% reduction in ER-mediated remodeling and formation of replicative LCVs (47). However, the *ylfA-ylfB* double mutant is similar to the WT strain in lysosomal evasion, indicating that ER-mediated remodeling and lysosomal evasion of the LCV are two independent events (47). However, the role of this LegC7/LegC2/LegC3 complex in association with MavE in intracellular proliferation and lysosomal evasion by L. pneumophila is not known.

The combination of the eukaryotic motifs in MavE and metaeffector activity of YlfA/LegC7 suggests the MavE effector is likely involved in governing biogenesis of the nascent LCV (19). Here we demonstrate that the MavE effector is the first effector identified to be indispensable for diverting the LCV from the endosomal-lysosomal pathway and is essential for intracellular replication in human macrophages and amoebae, as well as for intrapulmonary proliferation in mice. MavE is localized to the LCV pole, which is consistent with localization of the Dot/Icm translocation apparatus. The crystal structure of MavE shows a single-pass transmembrane domain at its C terminus, an NPxY eukaryotic motif, and three copies of the tyrosine-based sorting motifs (48–50). Our data show the NPxY eukaryotic motif specifically the P and the upstream D residue of this motif are required for its function in ER-mediated remodeling of the LCV and lysosomal evasion, both of which are essential for intracellular proliferation.

## RESULTS

### Subcellular localization of MavE.

**FIG 1:**
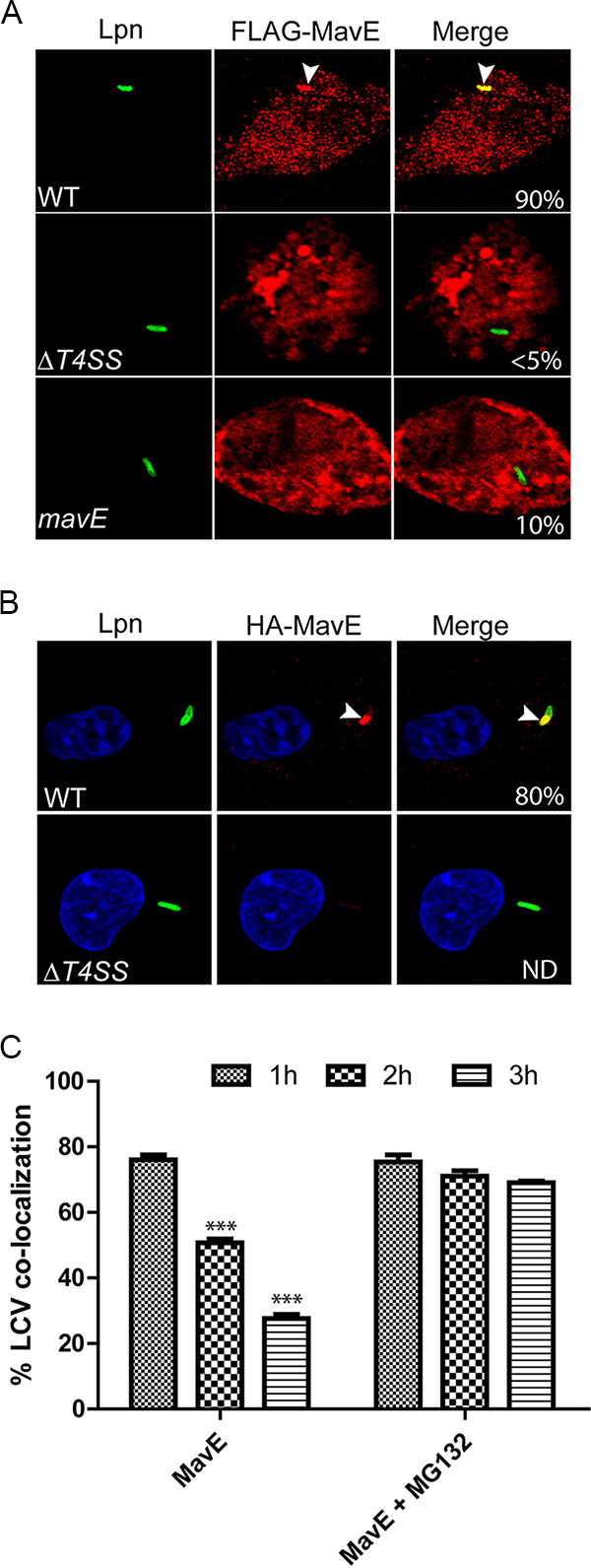
Localization of MavE during ectopic expression and during infection of hMDMs. (A) Representative confocal microscopy images of transfected HEK293T cells with 3×-FLAG-tagged MavE. Cells were infected for 2 h, in triplicate, using wild-type L. pneumophila, the *ΔT4SS* mutant, or the *mavE* mutant (green). Ectopic expression of MavE exhibited distinct punctate distribution throughout the cell (red). Quantification of colocalization of MavE with the LCV (indicated by arrowheads) (yellow) is shown in merged images. (B) Representative confocal microscopy images of 4HA-tagged MavE (red) constructs in both wild-type and *T4SS* L. pneumophila (green), following 1-h infection in hMDMs. Quantification of colocalization of MavE with the LCV (indicated by arrowheads) (yellow) is shown in merged images. ND, not detectable. (C) The presence of MavE on wild-type LCVs decreased from 1 h to 3 h postinfection. The proteasome inhibitor MG132 blocked the loss of MavE. Values that are significantly different by Student’s *t* test are indicated as follows: ***, *P ≤ *0.001.

Since pathogenic effectors injected into host cells have distinct subcellular localization where they interact with specific targets, the subcellular localization of MavE during infection was determined by transiently transfecting HEK293T cells with a construct encoding 3×-FLAG-tagged MavE to enhance expression and detection of MavE. Following 24 h, the cells were infected with wild-type L. pneumophila, a translocation-deficient (Δ*T4SS*) *dotA* mutant, or the *mavE* mutant for 2 h. Ectopic expression of MavE exhibited distinct punctate distribution throughout the cell, indicative of vesicular localization (Fig. 1A). Remarkably, 3×-FLAG-tagged MavE was trafficked to 90% of wild-type strain-containing LCVs, and this colocalization is Dot/Icm dependent, since the Δ*T4SS* mutant vacuoles exhibited less than 5% colocalization. In cells infected with the *mavE* mutant, MavE was trafficked to 10% of LCVs only. These data suggest that other Dot/Icm-injected effectors may be required during infection, and injection of various effectors is required for proper localization of MavE to the LCV membrane.

To examine subcellular localization of MavE during infection of macrophages, we constructed L. pneumophila strains that express four hemagglutinin (4HA)-tagged MavE (4HA-MavE) fusions and infected human monocyte-derived macrophages (hMDMs) for 1 h. Following fixation, the plasma membranes of infected hMDMs were differentially permeabilized using digitonin to detect whether MavE was exposed to the cytosol. The 4HA-MavE translocated by wild-type bacteria was spatially and exclusively localized to the cytosolic side of 80% of the LCVs (Fig. 1B). Interestingly, MavE was concentrated at the LCV poles, which is consistent with the localization of the Dot/Icm translocation system to the bacterial poles (51). MavE was not detected on LCVs harboring the Δ*T4SS* mutant (Fig. 1B). To exclude the potential effect of digitonin on colocalization to the LCV, methanol fixation was utilized after the infection without digitonin. The data showed that the 4HA-tagged MavE was consistently concentrated at the LCV poles in hMDMs infected by the WT strain in a Dot/Icm-dependent manner (see Fig. S1 in the supplemental material).

10.1128/mBio.03458-20.1 FIG S1 Localization of MavE during infection of hMDMs without digitonin treatment. (A) Representative confocal microscopy images of 4HA-tagged MavE (red) constructs in both the wild type and Δ*T4SS* mutant of L. pneumophila (green), following 1-h infection in hMDMs followed by methanol fixation without digitonin treatment. ND indicates not detected. Quantification of colocalization with the LCV (indicated by arrowheads) (yellow) is shown in merged images. Infected monolayers were fixed in −20°C methanol for 5 min. The results are representative of three independent experiments performed in triplicate. Download FIG S1, PDF file, 0.2 MB. Copyright © 2021 Vaughn et al. 2021 Vaughn et al. This content is distributed under the terms of the Creative Commons Attribution 4.0 International license.

The detection of MavE on wild-type strain-containing LCVs decreased rapidly and significantly over time, with only 30% of LCVs decorated with MavE at 3 h postinfection. As the HA-tagged MavE used was isopropyl-β-d-thiogalactopyranoside (IPTG) inducible, the MavE was synthesized by the bacteria prior to infection. To determine whether the loss of MavE was due to host proteasomal degradation, MG132 was used to inhibit the proteasomes (52). We have shown previously that inhibition of proteasomal degradation blocks replication of L. pneumophila within hMDMs due to the lack of sufficient amino acids but has no impact on phagosomal trafficking and is totally reversible upon supplementation of amino acids (52). The data showed that upon inhibition of the proteasomes, MavE was retained on the LCVs, suggesting that MavE is degraded by the host proteasomes (Fig. 1C).

### Role of MavE in intracellular replication.

**FIG 2:**
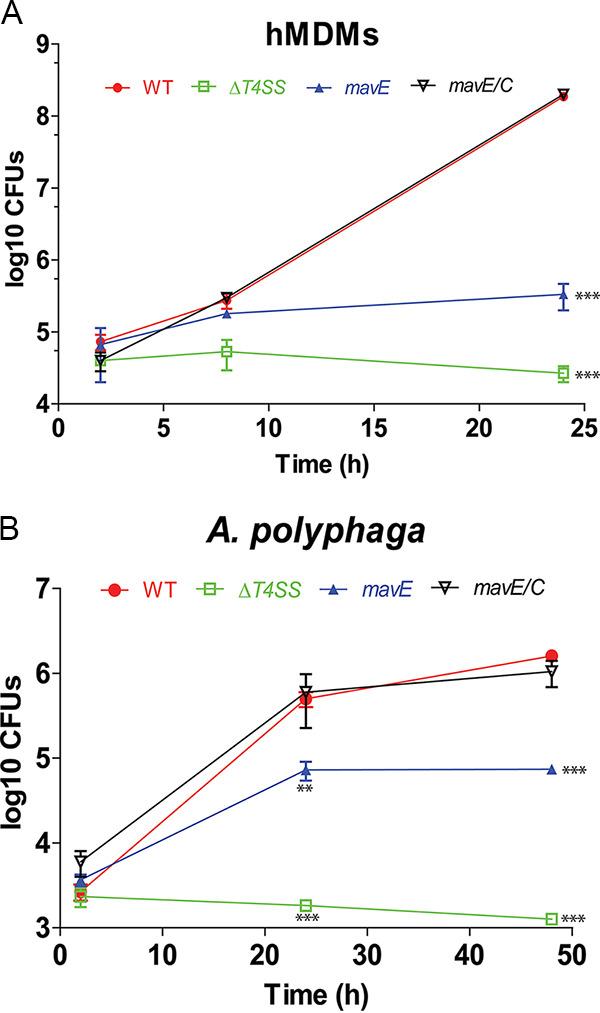
MavE is essential for intracellular replication in hMDMs and *A. polyphaga.* (A) To determine intracellular replication of the WT strain, the *T4SS* mutant, the *mavE* mutant, and complemented *mavE* mutant (*mavE*/*C*), hMDMs were infected, and the number of CFU was determined at 2 and 24 h postinfection. Data points represent mean CFU ± standard deviations (SD) (error bars) and are representative of three independent experiments. (B) To determine intracellular replication of the WT strain, the *T4SS* mutant, the *mavE* mutant, and complemented *mavE* mutant (*mavE*/*C*), *A. polyphaga* was infected, and the number of CFU was determined at 2, 24, and 48 h postinfection. Data points represent mean CFU ± SD and are representative of three independent experiments. Statistical significance by Student’s *t* test is indicated as follows: **, *P ≤ *0.01; ***, *P ≤ *0.001.

With few exceptions, most single effector mutants in L. pneumophila do not exhibit a defective phenotype in macrophages (7). To further examine the function of MavE, we determined whether MavE was required for intracellular replication. An isogenic mutant was generated and used to infect hMDMs. Growth of the *mavE* mutant during *in vitro* broth culture was identical to the wild-type strain (Fig. S2A). The data showed that the *mavE* mutant failed to replicate in hMDMs, similar to the translocation-deficient *T4SS* mutant (Fig. 2A). Complementation of the *mavE* mutant (*mavE*/*C*) reversed the severe intracellular growth defect. Infection of Acanthamoeba polyphaga, a natural host of L. pneumophila, exhibited ∼10-fold-less *mavE* mutant bacteria recovered at 24 and 48 h postinfection compared to those infected with the wild type or the complemented mutant (*mavE*/*C*) (Fig. 2B).

10.1128/mBio.03458-20.2 FIG S2 Growth of substitution mutants *in vitro* and expression and stability of the variant MavE proteins. (A) Overnight cultures of WT and Δ*mavE* strains in BYE broth were grown overnight at 37°C, diluted to an OD_550_ of 0.05, and grown at 37°C for 24 h. Growth rates were determined by measuring optical density at 550 nm every 2 h for 12 h and then again at 24 h postinoculation. Data are representative of three independent experiments. (B) Overnight cultures of WT, Δ*mavE*, and NPxY substitution mutants in BYE broth were grown overnight at 37°C, diluted to OD_550_ of 0.05, and grown at 37°C for 24 h. Growth rates were determined by measuring optical density at 550 nm every 2 h for 12 h and then again at 24h postinoculation. Data are representative of three independent experiments. (C) Immunoblots of total bacterial lysates from WT L. pneumophila and each of the MavE substitution attenuated mutant strains. Cell lysates of equivalent numbers of bacteria were subjected to immunoblotting using rabbit anti-MavE antiserum (104, 111). Download FIG S2, PDF file, 0.6 MB. Copyright © 2021 Vaughn et al. 2021 Vaughn et al. This content is distributed under the terms of the Creative Commons Attribution 4.0 International license.

**FIG 3:**
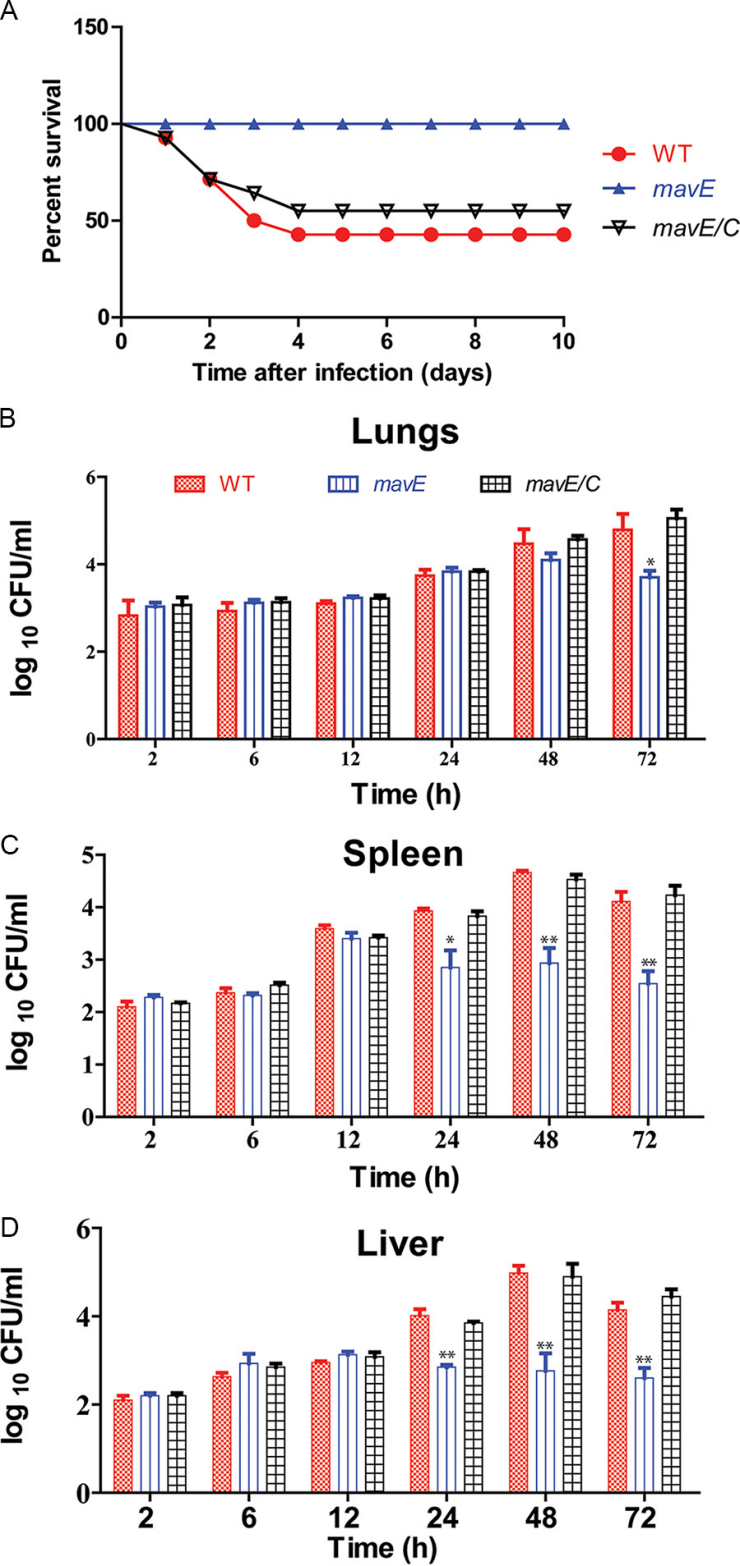
The MavE effector is essential for intrapulmonary replication *in vivo*. (A) Mouse lethality assay in which A/J mice were intratracheally infected with the wild type, the *mavE* mutant, and complemented strains (*mavE*/*C*) at 10^7^ CFU and monitored for 10 days. (B) Lung CFU were determined in A/J mice at 2, 6, 12, 24, 48, and 72 h postinoculation. The CFU in the *mavE* mutant intratracheally infected mice compared to both the wild type and complemented strains (*mavE*/*C*) are shown. (C) Spleen CFU of the wild type, *mavE* mutant, and complemented strain (*mavE*/*C*) were determined in intratracheally infected A/J mice at 2, 6, 12, 24, 48, and 72 h postinoculation. (D) Liver CFU were determined in intratracheally infected A/J mice at 2, 6, 12, 24, 48, and 72 h postinoculation for the wild type, *mavE* mutant, and complemented strain (*mavE*/*C*). Statistical significance by Student’s *t* test is indicated as follows: *, *P ≤ *0.05; **, *P ≤ *0.01.

In the A/J mouse lethality model, using an intratracheal inoculation of 10^7^ CFU (50% lethal dose [LD_50_]), the *mavE* mutant was completely attenuated, with 100% animal survival at 10 days postinfection (Fig. 3A). In contrast, both the WT and the complemented *mavE* mutant showed 50% lethality (Fig. 3A). To determine whether the *mavE* mutant has a defect in intrapulmonary growth, A/J mice were intratracheally infected with 10^6^ CFU to determine intrapulmonary proliferation. At 2, 6, 12, 24, 48, and 72 h, the bacterial burden was determined in the lungs, spleen, and liver. Compared to the WT bacterial burden, a decrease of bacterial burden of the *mavE* mutant was observed in lung tissue at 72 h postinfection (Fig. 3B). Following 24 h postinfection, a decrease in bacterial burden was found in both the spleen and liver for the *mavE* mutant bacterial strain (Fig. 3C and D). The A/J mice intratracheally infected with the complemented *mavE* mutant and mice infected by WT bacteria exhibited similar bacterial burdens in the lungs, spleen, and liver. Taken together, the MavE effector is essential for intracellular growth in macrophages, amoeba and for intrapulmonary proliferation in mice.

### Role of MavE in ER-mediated remodeling and lysosomal evasion by the LCV.

**FIG 4:**
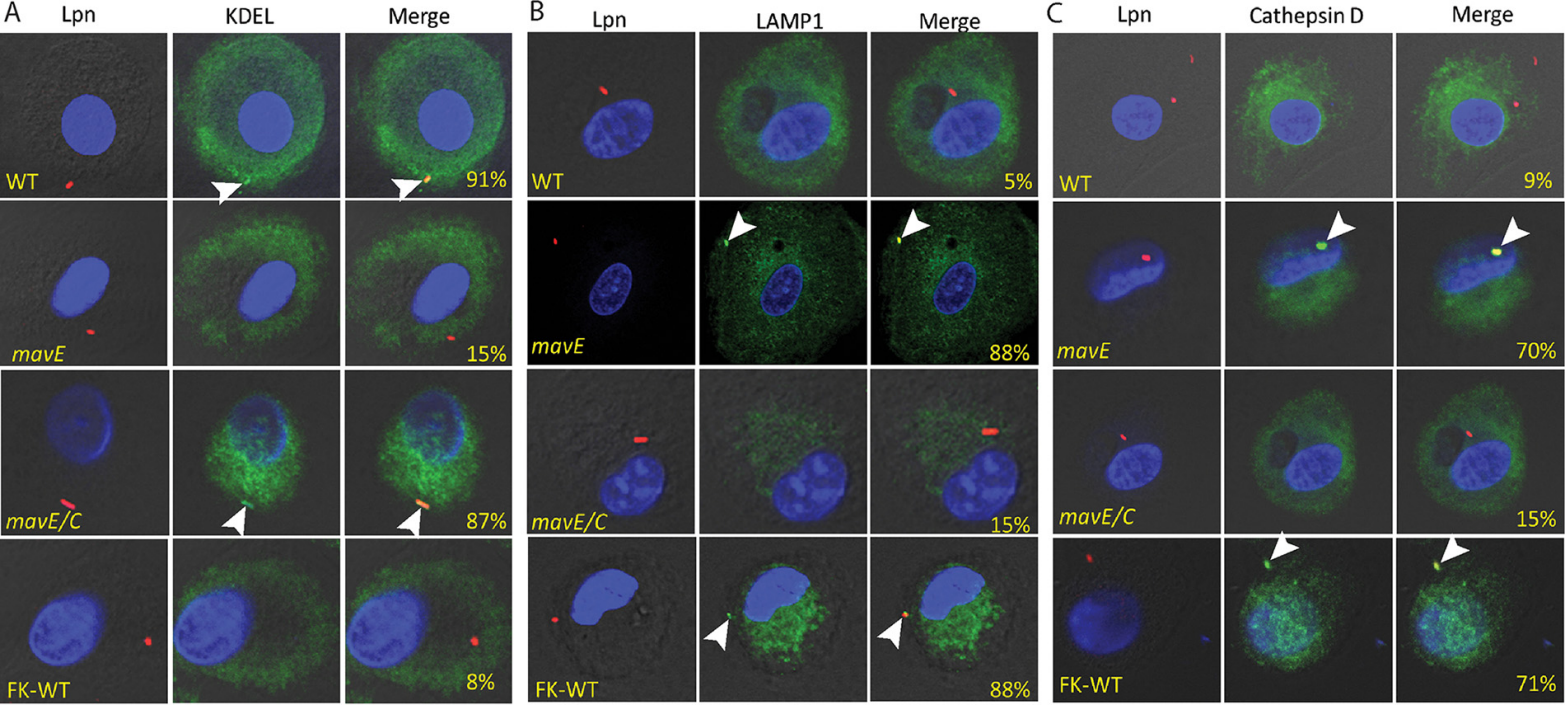
Fusion of the vacuoles containing the *mavE* mutant with the lysosome. (A) Colocalization of the LCVs containing the wild type, formalin-killed (FK) L. pneumophila, *mavE* mutant, or the complemented strain (*mavE*/*C*) labeled with the ER marker, KDEL (green), DAPI (blue), and anti*-Legionella* (red). Quantification of colocalization with the LCV (indicated by arrowheads) (yellow) is shown in merged images. (B) Colocalization of the LCVs containing the wild type, formalin-killed (FK) L. pneumophila, *mavE* mutant, or the complemented strain (*mavE*/*C*) stained with the late endosome/early lysosomal marker, LAMP1 (green), DAPI (blue), and anti*-Legionella* (red). Quantification of colocalization with the LCV (indicated by arrowheads) (yellow) is shown in merged images. (C) Colocalization of the LCVs containing the wild type, formalin-killed L. pneumophila, *mavE* mutant, or the complemented strain (*mavE*/*C*) stained with the lysosomal marker, Cathepsin D (green), DAPI (blue), and anti*-Legionella* (red). Quantification of colocalization with the LCV (indicated by arrowheads) (yellow) is shown in merged images. All analyses were performed on 100 infected cells analyzed from multiple coverslips. The results are representative of three independent experiments performed in triplicate.

Since the *mavE* mutant is defective for intracellular growth, we determined whether trafficking of the vacuole harboring the mutant was altered. To determine whether the *mavE* mutant failed to create an ER-derived vacuole, we utilized confocal microscopy to determine colocalization of the LCV with the ER marker, KDEL. The data showed that over 90% of LCVs harboring wild-type bacteria colocalized with KDEL, while only 15% and 8% of the *mavE* mutant and formalin-killed WT (FK-WT)-containing vacuoles, respectively, colocalized with the ER marker (Fig. 4A). Much like WT-containing LCVs, 87% of the complemented mutant (*mavE*/*C*) containing LCVs colocalized with KDEL.

To examine colocalization with late endosomes/lysosomes, the late endosome/lysosomal marker, LAMP1, was utilized. Our data showed that only 5% of the wild-type bacteria-containing LCVs colocalized with the LAMP1 marker. In contrast, 88% of LCVs harboring the *mavE* mutant colocalized with LAMP1 (Fig. 4B). The defect of the mutant was restored upon complementation where only 15% of the vacuoles harboring the complemented *mavE* mutant colocalized with LAMP1. For the LCVs containing FK-WT bacteria as a control, ∼90% of the vacuoles colocalized with the LAMP1 marker.

**FIG 5:**
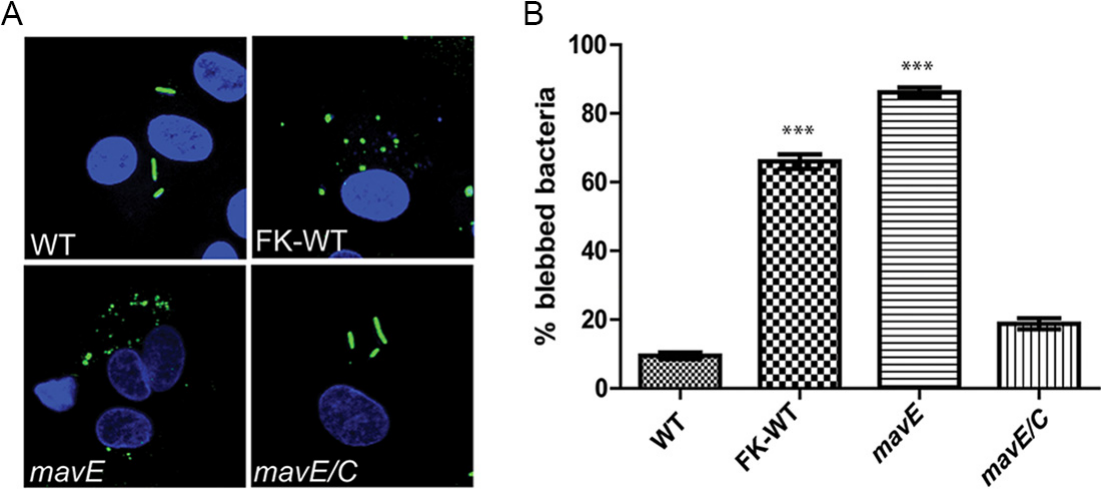
An essential role for MavE in bacterial viability within the LCVs of hMDMs. (A) Representative confocal microscopy images of the wild type, formalin-killed wild type, *mavE* mutant, and the translocation-deficient *T4SS* mutant L. pneumophila, following 1- h infection in hMDMs. The cells were fixed and stained with anti-*Legionella* (green) and DAPI (blue). All analyses were performed on 100 infected cells analyzed from multiple coverslips. The results are representative of three independent experiments performed in triplicate, and error bars represent standard deviations. ***, *P ≤ *0.001 by Student’s *t* test.

Importantly, 70% of LCVs harboring the *mavE* mutant colocalized with the lysosomal marker, Cathepsin D, similar to formalin-killed bacteria, while only 9% of LCVs harboring the wild-type strain localized with Cathepsin D (Fig. 4C). Importantly, in contrast to the bacillus shape of WT bacteria, the majority of the *mavE* mutant bacteria exhibited altered morphology that included rounding and blebbing, indicative of bacterial degradation, which is consistent with fusion of the LCV to the lysosome (Fig. 5). Taken together, MavE is the first effector of L. pneumophila shown to be indispensable for biogenesis of the LCV and lysosomal evasion.

### Crystal structure of MavE.

**FIG 6:**
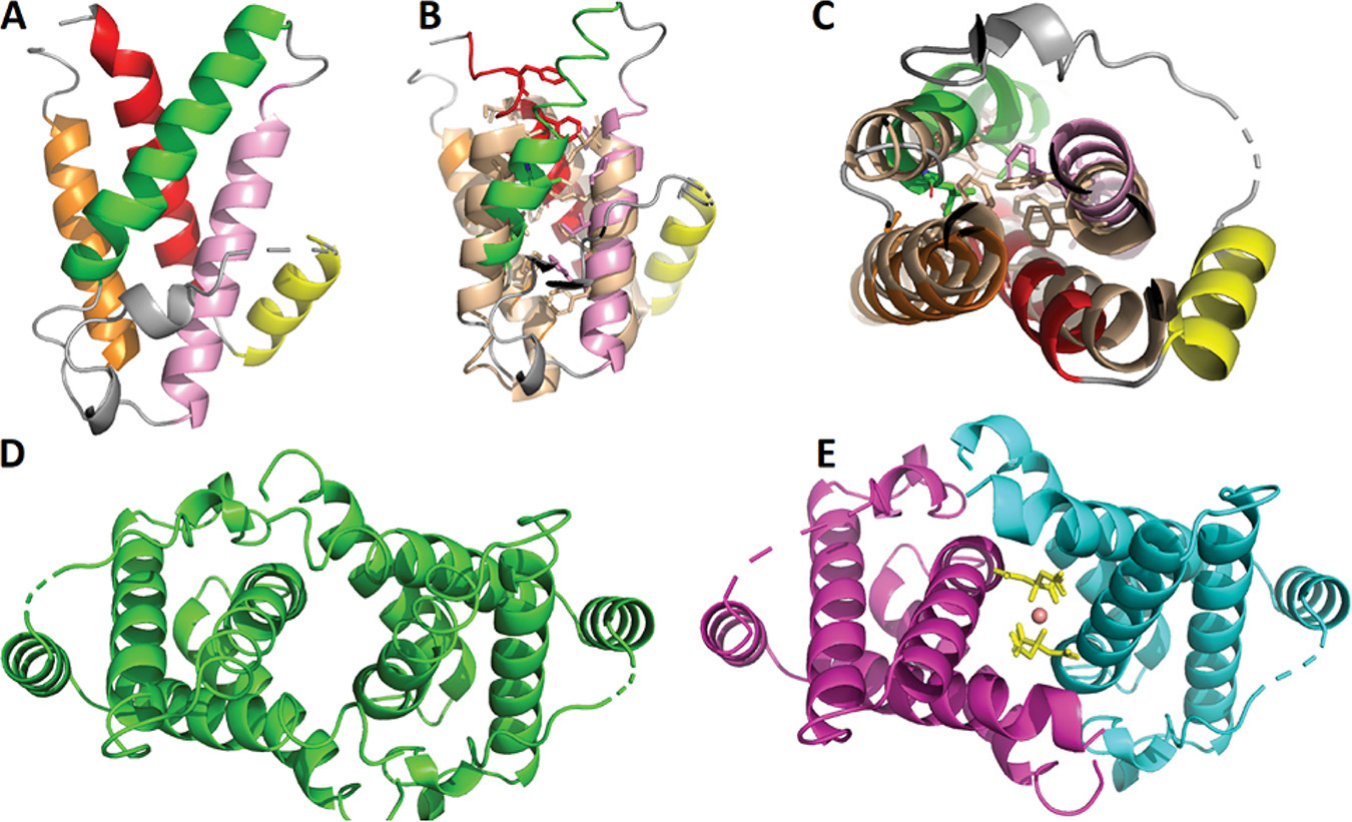
Crystal structure of MavE (39-172). (A) Overall structure of a single MavE chain. Helices A, B, C, D, and E are colored red, yellow, pink, green, and orange, respectively. Loops are shown in gray. (B) Overlay of MavE (39-172) and the grass pollen allergen Phlp 5b. MavE is colored as in panel A, and the grass pollen allergen is colored wheat. Hydrophobic core residues are depicted as sticks. (C) View of the overlay shown in panel B from below. Note that helix E and the loop connecting it to helix B are absent in Phlp 5b. The dimer interface for AA and BC chains is depicted in panels D and E, respectively.

MavE contains a predicted transmembrane helix at the C terminus (amino acids [aa] 183 to 206). This region was excluded from the constructs submitted to crystallization to ensure protein solubility. We succeeded in crystallizing construct MavE (39-172 [aa 39 to 172]). Its crystal structure was solved by single anomalous dispersion (SAD) and refined to a resolution of 1.8 Å. There are three molecules in the asymmetric unit, designated *A*, *B*, and *C*. Molecules *B* and *C* are related by twofold symmetry, and molecule *A* is also related by a twofold axis to a *B* chain in a neighboring unit cell (Fig. 6A). This organization results in two layers of *B* and *C* molecules being sandwiched between a single layer of *A* molecules (Fig. 6A). MavE (39-172) is comprised of five α-helices, which are designated from the N to C terminus as A, B, C, D, and E. The N- and C-terminal residues of MavE are near one another, with helices A and E making contacts via Arg155 and Arg162 guanidinium nitrogen atoms in E (Arg155/162) forming hydrogen bonds to Leu57 carbonyl oxygen and Glu54 side chain oxygen atoms in A, respectively. Helices B and C are connected by a long (22-residue) loop, with the first ∼10 residues (Gln72-Arg79) having poorly defined electron density. The N^77^PxY^80^ motif, which is disordered in the structure, is located within this loop and stretches across helices C and D which, in turn, lie diagonally overtop helices A and E. Thus, helices A and E act as a narrow scaffold upon which helices B, C, and D are positioned, with the longest loop of the structure (between helices B and C) rendered solvent accessible.

Significant intermolecular contacts in the MavE crystal occur at the *AA* or *BC* dimer interfaces. Each ∼1,000-Å^2^ interface is formed by N- and C-terminal residues, which contact a kinked region of helix C in the symmetry-related molecule. Two centrally positioned citrate molecules strengthen the *BC* dimer interaction using a water-mediated hydrogen bond network. Residues contributing to this interaction are Ala38, Glu42, Gln125, and Ser128 of each chain. The difference electron density map (mFo − DFc) indicates the presence of another stabilizing element at the *A-A* interface, although the identity of this small molecule remains unclear.

To gain insight into MavE function, we searched for proteins with a similar fold. To this end, we submitted MavE (39-172) coordinates to the DALI server (53). The most interesting result in our search for homologues was a core domain of the grass pollen allergen, Phlp 5b (PDB identifier [id] 1L3P, Z-score = 7.1). This domain is comprised of a four-helix bundle (54). Helices A, B, C, and D of MavE (39-172) align well with the Phlp 5b core domain (Fig. 6B), but helix E has no counterpart in Phlp 5b. Two 35-residue helix-turn-helix motifs are present in Phlp 5b, and they share 37% sequence identity with one another. These motifs adopt remarkably similar helix termination and chain reversal strategies. As such, Phlp 5b was described as a twinned two-helix bundle. The corresponding helix-turn-helix motifs in MavE also share several residues, with some stabilizing the core architecture. Based on these observations, MavE can be said to have a similar core domain to that of Phlp 5b. Interestingly, the extended loop between helices B and E in MavE is not present in Phlp 5b and constitutes an insertion into this fold. This insertion contains not only the NPxY motif but also a triad Asp64-His68-Ser102 that is reminiscent of the catalytic triad of serine proteases. Two of these residues are located within the insertion loop. While these side chains in the crystal structure are not connected by hydrogen bonds (as they are in the catalytic triad of proteases), they could be brought into such interactions by simple rotation of the sidechains (Fig. 6), suggesting a possibility that MavE possesses catalytic activity.

### Structure-based functional analysis of MavE.

**FIG 7:**
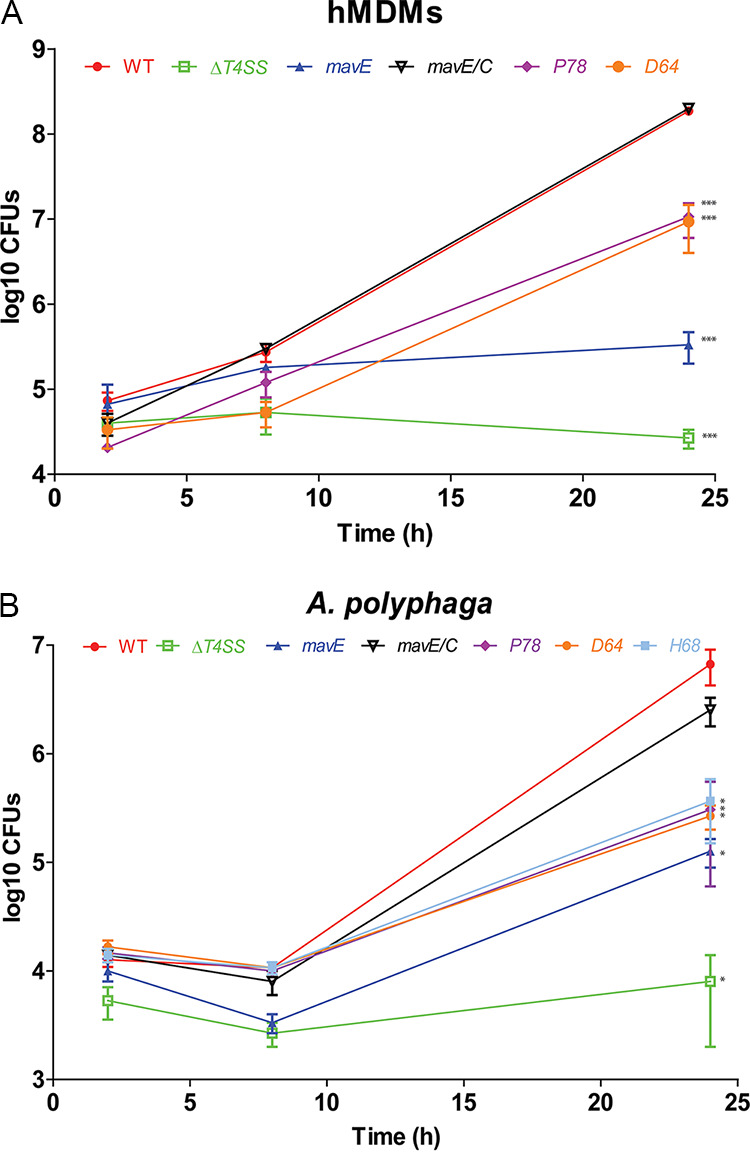
Role of the NPxY motif in biological function of MavE in hMDMs and *A. polyphaga*. (A and B) hMDMs (A) and *A. polyphaga* (B) were infected with the WT strain, the *T4SS* mutant, the *mavE* mutant, NPxY substitution mutants (D64A and P78A), and complemented *mavE* mutant (*mavE*/*C*), and the number of CFU were determined at 2, 8, and 24 h postinfection. Data points represent mean CFU ± SD and are representative of three independent experiments. Data points represent mean CFU ± SD and are representative of three independent experiments. Statistical significance by Student’s *t* test is indicated as follows: *, *P* ≤ 0.05; ***, *P ≤ *0.001.

To determine the role the NPxY motif has in the function of the MavE effector during infection, six point mutations were made in the critical domains predicted to contribute to the biochemical function of MavE. Three residues in the NPxY motif (N77A, P78A, and Y80A) were targeted for substitution. They are located on the insertion loop, are exposed on the MavE surface, and show high mobility/flexibility (poor electron density). Three other residues (D64-H68-S102), which constituted a potential catalytic triad reminiscent of the catalytic triad of serine proteases, were also targeted for substitution mutagenesis. Growth of the *mavE* substitution mutants during *in vitro* broth culture grew identical to the wild-type strain (Fig. S2B). The L. pneumophila expressing *mavE* variants were used to infect hMDMs to evaluate intracellular growth kinetics (Fig. S3). Two of the substitution mutants, D64A and P78A, were found to be attenuated (Fig. 7A). Similar results were observed in A. polyphaga. However an additional substitution mutant, H68A, also showed attenuation in *A. polyphaga* (Fig. 7B). Using immunoblots of total bacterial lysates, there was no major differences in the expression and stability of the variant proteins in the L. pneumophila variants with defective phenotypes (Fig. S2C). Thus, the structure of the MavE protein harbors several functional motifs, including an NPxY eukaryotic motif, and these motifs are required for the function of MavE in intracellular proliferation of L. pneumophila.

10.1128/mBio.03458-20.3 FIG S3 Role of the NPxY motif in biological function of MavE in hMDMs and *A. polyphaga*. (A) hMDMs and (B) *A. polyphaga* were infected to determine intracellular replication of the WT strain, the Δ*T4SS* mutant, the *mavE* mutant, NPxY substitution mutants (D64A, P78A, S102A, Y80A, H68A, and N77A), the WT strain harboring the pBCsk vector, and complemented *mavE* mutant (*mavE*/*C*) hMDMs. The number of CFUs was determined at 2, 8, and 24 h postinfection. Data points represent mean CFUs ± SD and are representative of three independent experiments done in triplicate. Statistical significance by Student’s *t* test: *, *P ≤ *0.05; **, *P ≤ *0.01; ***, *P ≤ *0.001. Download FIG S3, PDF file, 0.9 MB. Copyright © 2021 Vaughn et al. 2021 Vaughn et al. This content is distributed under the terms of the Creative Commons Attribution 4.0 International license.

**FIG 8:**
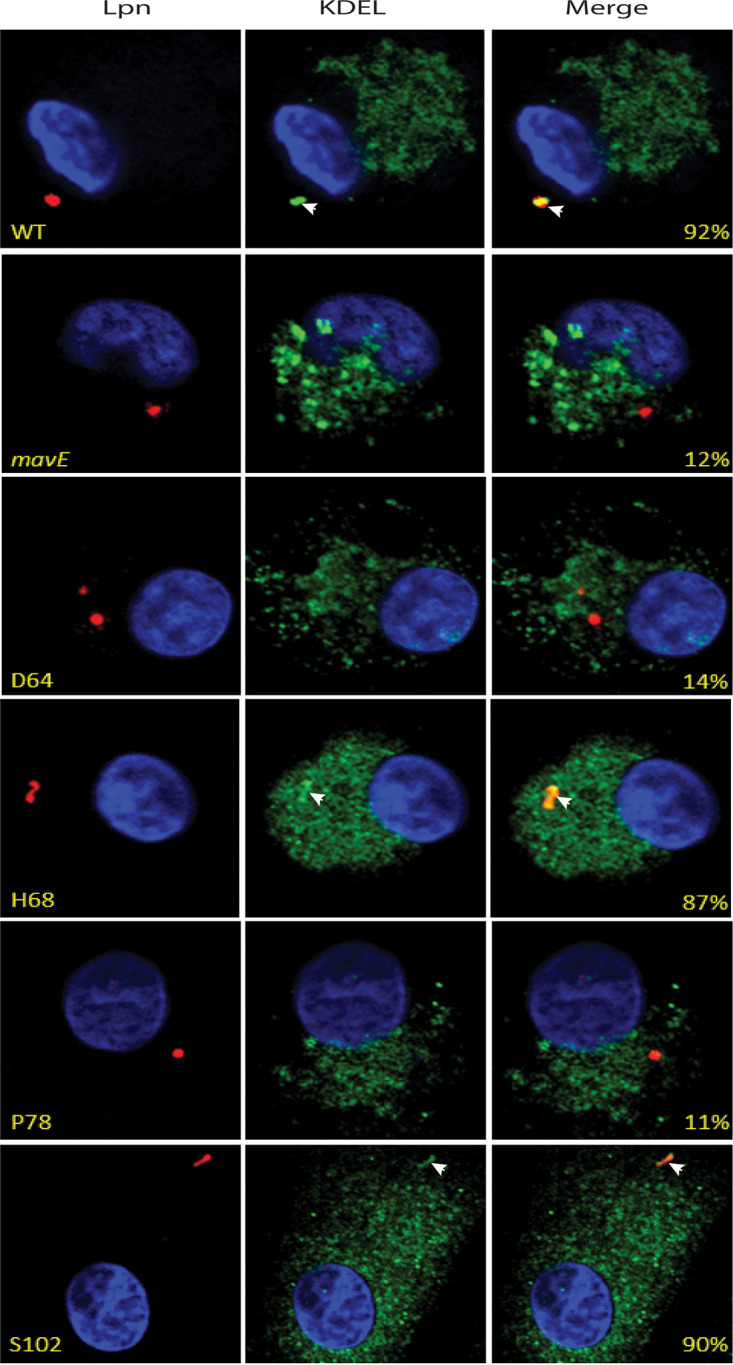
Remodeling of the vacuoles with the ER. Representative confocal microscopy images of colocalization of the LCVs containing the wild type, *mavE* mutant, or NPxY substitution mutants. D64A, H68A, P78A, and S102A strains were labeled with the ER marker, KDEL (green), DAPI (blue), and anti*-Legionella* (red). Quantification of colocalization with the LCV (indicated by arrowheads) (yellow) is shown in merged images. All analyses were performed on 100 infected cells analyzed from multiple coverslips. The results are representative of three independent experiments performed in triplicate.

To determine whether the *mavE* substitution mutants shown essential for intracellular replication in hMDMs also exhibit altered phagosome biogenesis, we utilized confocal microscopy to determine colocalization of the LCV with the ER marker, KDEL (Fig. 8). The additional substitution mutant, H68A, shown to be attenuated in *A. polyphaga* and substitution mutant, S102A, which did not show attenuation, were used as controls. The data showed that 92% of LCVs harboring wild-type bacteria colocalized with KDEL, while only 12% of the *mavE* mutant strain-containing vacuoles colocalized with the marker (Fig. 8). Much like the *mavE* mutant strain-containing LCVs, 14% of the D64A substitution mutant LCVs and 11% of the P78A substitution mutant LCVs colocalized with KDEL. For the controls, 87% of the H68A substitution mutant and 90% of the S102A substitution mutant containing LCVs colocalized with the ER marker KDEL.

**FIG 9:**
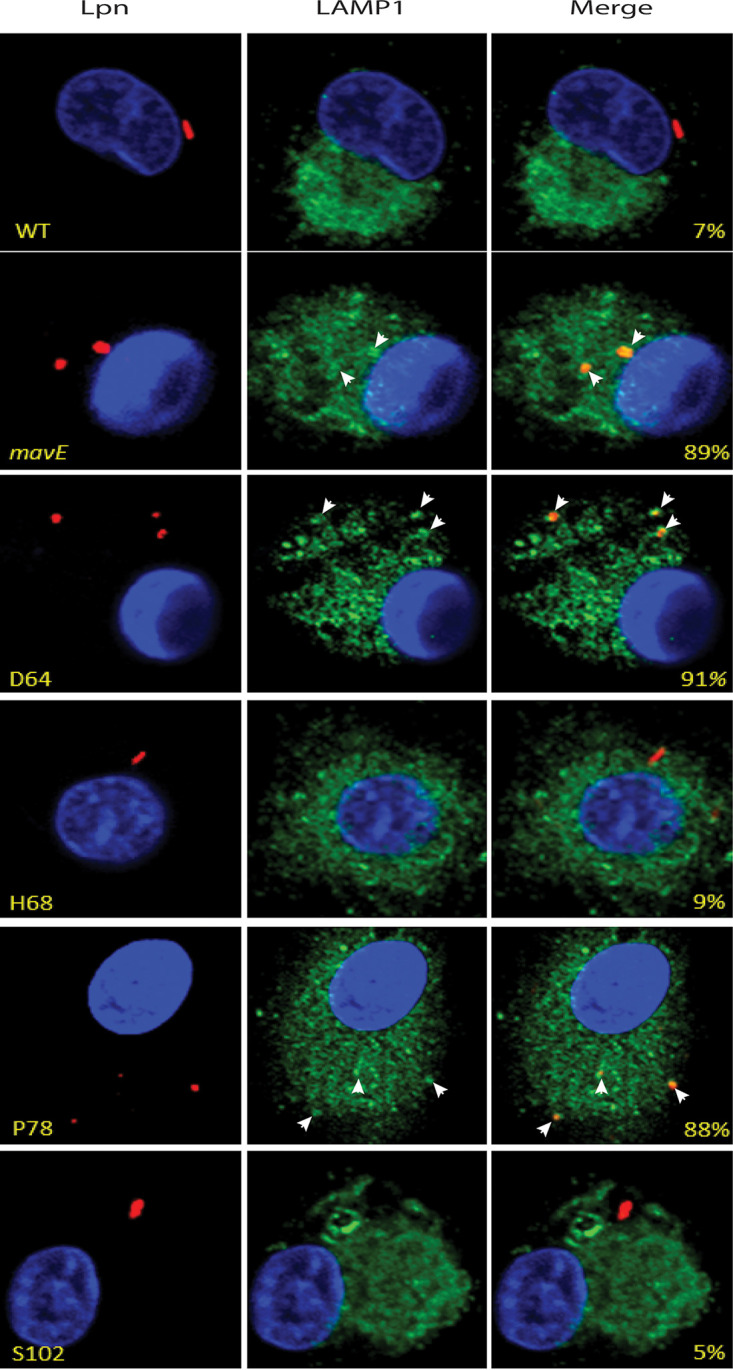
Fusion of the vacuoles containing the *mavE* substitution mutants with the late endosomes/lysosomes. Colocalization of the LCVs containing the wild type, *mavE* mutant, or the NPxY substitution mutants, D64A, H68A, P78A, and S102A strains with the late endosome/lysosomal marker, LAMP1 (green), DAPI (blue), and anti*-Legionella* (red). Quantification of colocalization with the LCV (indicated by arrowheads) (yellow) is shown in merged images. All analyses were performed on 100 infected cells analyzed from multiple coverslips. The results are representative of three independent experiments performed in triplicate.

Importantly, ∼90% of the LCVs containing the P78A and D64A substitution mutant bacteria colocalized with the LAMP1 marker, similar to the *mavE* mutant, while only 7% of the wild-type bacteria-containing LCVs colocalized with the marker (Fig. 9). The LCVs of the controls, S102, and the H68A substitution mutants showed 5 to 9% colocalization with LAMP1.

**FIG 10:**
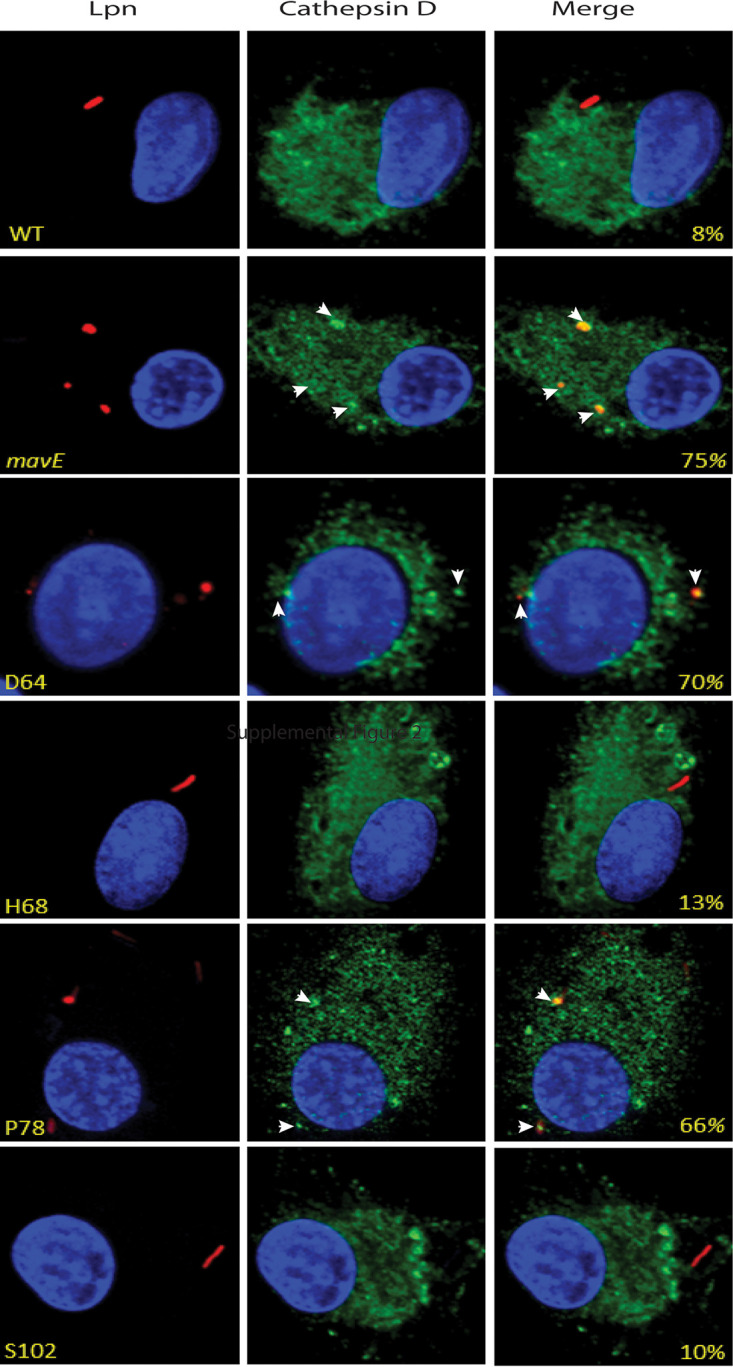
Fusion of the vacuoles containing the *mavE* substitution mutants with the lysosomes. Colocalization of the LCVs containing the wild type, *mavE* mutant, or the NPxY substitution mutants, D64A, H68A, P78A, and S102A strains with the lysosomal marker, Cathepsin D (green), DAPI (blue), and anti*-Legionella* (red). Quantification of colocalization with the LCV (indicated by arrowheads) (yellow) is shown in merged images. All analyses were performed on 100 infected cells analyzed from multiple coverslips. The results are representative of three independent experiments performed in triplicate.

Our data showed that the D64A substitution mutant showed 70 to 75% colocalization with Cathepsin D, similar to the *mavE* mutant (Fig. 10). Importantly, 66% of the LCVs containing P78A substitution mutant colocalized with Cathepsin D, similar to the *mavE* null mutant (Fig. 10). Only 8% of LCVs harboring the wild-type strain localized with the lysosomal marker Cathepsin D. Only 10 to 13% of the LCVs containing the H68A and S102A substitution mutants colocalized with Cathepsin D. Importantly, similar to the *mavE* mutant, the majority of the P78A and D64A substitution mutant bacteria exhibited altered morphology with exaggerated rounding and blebbing, indicative of bacterial degradation and consistent with fusion of the LCV to the lysosomes. Taken together, the two substitution mutants, D64A and P78A, phenocopied the *mavE* null mutants in the failure to create ER-derived vacuoles that bypass the lysosomal degradation pathway and to proliferate in hMDMs. Thus, the P78 residue within the NPxY motif and the upstream D64 residue are required for the function of MavE in phagosome biogenesis and lysosomal evasion.

## DISCUSSION

Intracellular pathogens, whether cytosolic or intravacuolar within macrophages, have evolved idiosyncratic mechanisms to evade various innate defense processes avoiding degradation within macrophages (55–58). These pathogenic mechanisms involve the injection or secretion of effectors into the host by various type I to IX translocation systems (59–62) and are present in extracellular pathogens, such as *Bordetella* (63) as well. These effectors modulate various cellular processes as well as host metabolism to render the host cell suitable as a proliferative nutrient-rich niche (55, 64–70). Cytosolic pathogens, such as *Rickettsia*, have evolved to evade the host cytosolic antimicrobial processes (71, 72). Modulation of host metabolism is a general common theme among intracellular pathogens, leading to a suitable nutritional niche for pathogen proliferation, and that has been well characterized for *Mycobacterium* (52, 65, 66, 73–75). For intravacuolar pathogens, the crux of these host processes is evasion of the endosomal-lysosomal degradation pathway by most intravacuolar pathogens, such as *Mycobacterium*, *Chlamydia*, and *Salmonella* (1, 2). Modulation of the macrophage autophagy (76) and M1 versus M2 polarization by intracellular pathogens (77) is an emerging theme to modulate the host inflammatory response (78, 79). The intravacuolar pathogen L. pneumophila resides within the LCV that seems to be excluded from the endocytic pathway and is more of an ER-like compartment than a phagosome (1, 11). While there are numerous studies on translocated effectors of L. pneumophila and few of them have a partial role in lysosomal evasion, none of the effectors are indispensable for lysosomal evasion (11, 80, 81). With few exceptions, single effector deletions for most characterized effectors of L. pneumophila do not exhibit a defective intracellular growth phenotype (82). Redundancy among the L. pneumophila effectors occurs in different manners, and deletion of redundant paralog effectors does not always impair intracellular growth (7, 23, 83). Here, we show MavE is the first effector to be indispensable for both phagosome biogenesis and lysosomal evasion by L. pneumophila. The altered LCV biogenesis in the absence of MavE could be an indirect by-product of lysosomal evasion. MavE is localized at the cytosolic side of the LCV poles and is likely functioning in subverting vesicular fusion with the endocytic pathway. It has been shown that polar localization is a key feature of virulence of L. pneumophila, and this polarity could result in an increased localized concentration of subdomains of certain effectors at the LCV pole, possibly meeting a critical threshold concentration necessary for effector activity (84). Although ER remodeling of the LCV and its lysosomal evasion are independent processes, it is not known which comes first (85). LCVs communicate with and modulate the secretory vesicle trafficking pathway; however, little is known about the temporal aspect of ER remodeling and lysosomal evasion by the LCV (85).

The MavE structure contains a core domain reminiscent of the grass pollen allergen Phlp 5b. Compared to allergen Phlp 5b, MavE contains a long insertion loop located between helix B and helix C. These insertions represent a region of functional significance to MavE, as their absence in Phlp 5b suggests it is dispensable for protein stability. It has been suggested that the compactness of the twinned two-helix core domain facilitates proteolytic resistance in Phlp 5b (54). In accordance with this notion, MavE may have evolved this domain to act as a robust scaffold for displaying functional elements to binding partners. The fact that Phlp 5b retains allergenicity can further rationalize this claim, as L. pneumophila effectors must resist innate immune defenses. This stability might be essential for effectors performing a critical function in virulence, especially those having no redundant counterpart.

Structural features outside the MavE core domain are of functional significance to this protein. The loop connecting helices B and C is the longest in our model and contains a region of conformational heterogeneity, as evidenced by high B-factors and the absence of clear electron density. Intriguingly, the B-C loop harbors an NPxY sequence, which is the canonical phosphotyrosine-binding (PTB) domain-interacting motif (86). The presence of this motif on a flexible, solvent-exposed loop in MavE may suggest a role for this protein in recruiting PTB domains, which predominantly exist on adaptor or scaffold proteins (49). The two substitution mutants, D64A and P78A, are essential for MavE function within hMDMs and *A. polyphaga*, with an additional mutant, H68A, to be essential in amoebae. Neither the N77A nor Y80A variant significantly impedes L. pneumophila replication within hMDMs, whereas all three NPxY motif variants are similarly attenuated in *A. polyphaga*. The attenuated replication of the D64A and H68A substitution mutants can be explained by disrupting the putative activity of MavE. However, it is plausible that certain substitutions of residues (D/H) for a nonpolar one (A) may alter the structure due to differences in electronegativity. Our findings highlight the significance of the NPxY motif, and specifically H68A, to the function of MavE and possible L. pneumophila divergence between human and amoeba hosts. The variation between essential MavE function within hMDMs and *A. polyphaga* of the substitution mutants may be due to the evolutionary distance between host cells. The evolution of amoeba host-specific effectors that modulate amoeba-specific cellular processes by L. pneumophila may explain why only two substitution variants of MavE result in loss of its function in biogenesis of the LCV in hMDMs.

The proline residue within NPxY has been shown to form a β-turn structure that distinguishes this motif from the similar NxxY sequence, which is also recognized by adaptor proteins (87). This structural distinction between NPxY and NxxY provides a source of specificity for adaptor proteins (88). Our results suggest that the NPxY motif of MavE has a β-turn structure that is critical for its interaction with host adaptors as well as a metaeffector(s). Further investigation of these host adaptors and other PTB domain-containing proteins that are selective for NPxY motifs will provide additional insight into the role of MavE.

The primary hosts of L. pneumophila are lower eukaryotes, such as free-living amoebae, which carry genes that encode a small number of PTB domain-containing proteins (89). Only two PTB domain-containing proteins are present in Dictyostelium discoideum, namely, Talin A and B (49). These proteins link the cytoplasmic domains of integrin β-subunits to actin filaments, and thereby promote the formation of cellular junctions with the extracellular matrix. Acanthamoeba castellanii, a natural host of L. pneumophila, also carries a gene that encodes a putative Talin protein (90). Since Talin is the only PTB domain-containing protein found in the natural hosts of *Legionella*, MavE may interfere with host processes requiring functional Talin (49). Specifically, the recruitment of Talin by MavE could disrupt the organization of host actin (91) and thus alter the standard progression of vacuolar biogenesis events. In this way, MavE may participate in evasion of LCV-lysosome fusion.

Helices B and C of MavE (39-172) contain S102, H68, and D64 in a negatively charged pocket on the protein surface. Although these residues do not conform to the hydrogen bond network characteristic of a serine protease catalytic triad (92), their divergence from this pattern may be an artifact of crystal packing. Indeed, conformational changes in the flexible loop connecting helices B and C may affect the orientation of these two helices relative to one another. In addition, the electron density of the loop connecting helices C and D is unclear, suggesting flexibility in this region. Since movement of this loop would affect the orientation of helix C, this too could contribute to structural changes required to form the canonical catalytic triad. It is possible that our C-terminal truncation produced conformational changes in the soluble domain of MavE, which widen the putative active site and render it noncatalytic. Alternatively, the catalytic triad containing Ser102, His68, and Asp64 found in MavE may not exhibit a protease catalytic activity. Further studies evaluating the proteolytic activity of MavE will be informative.

In summary, our data show that MavE is involved in the acquisition of ER-derived membranes by the LCV and in evasion of lysosomal fusion. It is likely that both functions depend on the NPxY motif located within a solvent-exposed loop on the MavE structure. By mediating interactions with adaptor proteins, the NPxY motif of MavE may coordinate LCV trafficking through interaction with host adaptor proteins and other L. pneumophila metaeffectors such as YlfA/LegC7. Because of the unusually large repertoire of effectors, it is likely that additional L. pneumophila effectors also function to facilitate lysosomal evasion by the LCV (21, 47, 93). These effectors are likely to function in concert with temporal coordination among the effectors to enable ER remodeling and lysosomal evasion of the LCV. Future studies identifying any key players that interact with MavE as a complex and function with MavE in lysosomal evasion will generate a broader understanding of the main virulence strategy utilized by L. pneumophila.

## MATERIALS AND METHODS

### Strains and cell lines.

Legionella pneumophila strain AA100/130b (ATCC BAA-74), and the T4SS-deficient mutant (*lspG*) were grown on BCYE agar (17). To generate an isogenic *mavE* deletion mutant, 2 kb of DNA flanking either side of the *mavE* gene was amplified using PCR using primers listed in Table 1 and cloned into the shuttle vector, pBCSK+ (Stratagene), resulting in pBCSK+*mavE*KO1. To delete the entire *mavE* gene within pBCSK-*mavE*KO1, inverse PCR was employed using primers listed in Table 1, resulting in pBCSK+*mavE*KO2. The kanamycin resistance cassette from the Ez-Tn*5* transposon was amplified using primers listed in Table 1, and the resulting PCR product was subcloned into pBCSK+*mavE*KO2 in between the *mavE*-flanking DNA regions using standard molecular biology procedures, resulting in pBCSK+*mavE*KO3. This plasmid was introduced into L. pneumophila AA100 via natural transformation, as described previously (94). Following 3 days, natural transformants were recovered by plating on BCYE agar supplemented with 50 μg/ml kanamycin. To confirm deletion of the *mavE* gene in the transformants, PCR was used using the primers listed in Table 1. To complement the *mavE* mutant, PCR was used to amplify the *mavE* gene and its upstream promoter region using primers listed in Table 1 and subcloned into pBCSK+, generating pBCSK+*mavE*/*C*. This plasmid was introduced into the *mavE* mutant via electroporation as described previously (95). Complemented *mavE* mutants were selected on BCYE plates supplemented with 5 μg/ml chloramphenicol, resulting in the complemented strain, *mavE*/*C*. For infections of cell monolayers, L. pneumophila was grown in BCYE agar plates with appropriate antibiotic selection at 37°C for 3 days prior to use in infections as described previously (96). Human monocyte-derived macrophages (hMDMs) were isolated from healthy donors and cultured in RPMI 1640 (Corning Cellgro) as described previously (96, 97). HEK293T cells (ATCC) were cultured in Dulbecco modified Eagle medium (DMEM) (Gibco) supplemented with 10% fetal bovine serum as previously described (96, 97). All methods were carried out and approved in accordance with the University of Louisville Institutional Review Board guidelines, and blood donors gave informed consent as required by the University of Louisville Institutional Review Board (IRB 04.0358).

**TABLE 1:** Primers used in this study^*a*^

Primer^*b*^	Orientation^*c*^	Sequence
Primers used to clone *mavE* and flanking DNA to generate the *mavE* mutant (pBCSK plasmid)		
MavE KO	F	GTCGACAGGTAATTTCTGATAATGAAC
MavE KO	R	TCTAGAATAGAGCCGTTGGAAGAAAGT
Primers used for inverse PCR to delete *mavE* from the above fragment to make the *mavE* mutant		
MavE KO	F	aaatttGTTTAAACGGAAGTGTTTACAGGATT
MavE KO	R	CCTGCAGGAGGTAGTGTTTTATACTAA
Primers used to clone into pBCSK to complement the *mavE* mutant		
MavE/C	F	AAGCTTATTATATAATGATTTATCAATTT
MavE/C	R	GGATCCTATTTGGTCCATCTTGAAC
Primers used to confirm KO of *mavE* in L. pneumophila		
MavE KO test	F	TTTTATATCTTTAGGTTCATTCA
MavE KO test	R	CTTGAAACGACCGTATTTG
Primers used for cloning into the p3XFLAG vector for eukaryotic expression		
3XFLAG mavE	F	AAGCTTCTGACTCGATTCATAATGCTTT
3XFLAG mavE	R	AGATCTTTATGGTTTGTTGCCAAACAAC
Primers used for cloning into the 4HA vector for bacterial expression		
HA MavE	F	ggatccCTGACTCGATTCATAATGC
HA MavE	R	aagcttTTATGGTTTGTTGCCAAACA

a All primers are 5′-phosphorylated.

b KO, knockout.

c F, forward; R, reverse.

### DNA manipulations.

DNA manipulations, generation of MavE substitutions, and restriction enzyme digestions were performed using standard procedures (97, 98). WT L. pneumophila expressing HA_4_-PieE was obtained from the MRC Centre for Molecular Bacteriology and Infection. The MavE construct replaced the PieE sequence using the same methods and restriction sites previously described (99). Restriction enzymes and T4 DNA ligase were purchased from NEB (Madison, WI). Plasmid preparations were performed with the PureLink HiPure Plasmid Maxiprep kit (Invitrogen). Purification of DNA fragments from agarose gels for subcloning was carried out with the QIAquick gel purification kit (Qiagen Inc., Valencia, CA).

### Intracellular replication in hMDMs and amoeba.

For infection of cell monolayers, L. pneumophila strains were grown in BYE broth with appropriate antibiotic selection, at 37°C with shaking, to post-exponential phase (optical density at 550 nm [OD_550_] of 2.1 to 2.2). *A. polyphaga* (ATCC) was cultured in PYG medium at 22°C, and experiments were performed in PY medium at 35°C as previously described (96). Human monocyte-derived macrophages were isolated from healthy donors and cultured in RPMI 1640 supplemented with 10% fetal bovine serum as previously described (96, 97).

The wild-type strain, *T4SS* and *mavE* isogenic mutants, and *mavE*/*C*, *mavE*/*Y80*, *mavE*/*H68*, *mavE*/*P78*, *mavE*/*N77*, *mavE*/*D64*, *mavE-pBCsk*, *mavE-4HA*, and *WT-pBCsk* complemented strains were grown to post-exponential phase in BYE broth at 37°C with shaking prior to infection and used to infect hMDMs and *A. polyphaga* as previously described (96, 97). A total of 1 × 10^5^ host cells were plated in 96-well plates and infected with L. pneumophila at a multiplicity of infection (MOI) of 10. The plates were centrifuged at 200 × *g* (5 min) to synchronize infection. After 1 h, cells were treated with gentamicin to kill extracellular bacteria as previously described (96, 97). Over a 24-h time course, host cells were lysed with sterile water (hMDMs) or 0.02% (vol/vol) Triton X-100 (*A. polyphaga*). L. pneumophila CFU were determined by plating serial dilutions onto BCYE agar.

### Transfection of HEK-293 cells (ATCC).

The *mavE* gene was cloned into the mammalian expression vector, p3XFlag-CMV-10 (Sigma). To generate the *mavE-^9^L^10^P*/*AA* allele, the wild-type p3XFlag-CMV-10 MavE plasmid was used as a template for PCR-based site-directed mutagenesis. HEK-293 cells (ATCC) were grown to 80% confluence and plated onto poly-l-lysine-treated coverslips in 24-well plates. Following 24 h of incubation, HEK293T cell monolayers were transfected with ∼2 μg of plasmid DNA encoding 3×-FLAG MavE/well by using polyethylenimine (Polysciences) for 24 h, following the manufacturer's recommendations (Roche) as previously described (100, 101).

### Mouse model.

For testing the virulence of the *mavE* mutant, specific-pathogen-free, 6- to 8-week-old A/J mice (Jackson) were used, as previously described (97, 102). Groups of three A/J mice, for each time point, were infected intratracheally with 1 × 10^6^ CFU. The wild-type strain, the *mavE* isogenic mutant, and complemented *mavE*/*C* strains were grown to post-exponential phase on BCYE plates at 37°C for 72 h prior to infection and used to infect A/J mice. At 2, 6, 12, 24, 48, and 72 h after infection, mice were humanely sacrificed, and their lungs, livers, and spleens were harvested and homogenized in sterile saline (5 ml), followed by cell lysis in distilled water. To determine CFU, serial 10-fold dilutions were plated on BCYE agar and incubated at 37°C for 72 h, and colonies were enumerated. The percent survival was recorded for groups of five A/J mice infected using an inoculation of 1 × 10^7^ CFU (LD_50_) for WT, *mavE*, and *mavE*/*C* strains from 0 to 10 days postinfection. All the experimental procedures were in accordance with national guidelines and were approved by the Institutional Animal Care and Use committee (IACUC) at the Faculty of Medicine, University of Rijeka. To determine any level of difference between groups, a value of “E” (the degree of freedom of analysis of variance or ANOVA), which should lie between 10 and 20 was determined to be 12 based on our sample size. (E = total number of animals − total number of groups).

### Confocal microscopy.

Processing of infected cells for confocal microscopy was performed as we described previously (97). 4HA-tagged MavE constructs in both wild-type and *T4SS* L. pneumophila were analyzed for confocal microscopy following 1-h infection in hMDMs. Colocalization of the LCVs containing WT, FK-WT, and *mavE* strains and *mavE*/*C*, *mavE*/*Y80*, *mavE*/*H68*, *mavE*/*P78*, *mavE*/*N77*, *mavE*/*D64*, *mavE-pBCsk*, and *WT-pBCsk* complemented strains were analyzed for confocal microscopy following 2-h infection in hMDMs. Cells were prepared using the same protocol for intracellular replication except 2 × 10^5^ host cells were plated on coverslips in 24-well plates. The monolayers were infected with L. pneumophila at an MOI of 10. Plates were centrifuged at 200 × *g* (5 min) to synchronize infection. After 1 h, cells were treated with gentamicin to kill extracellular bacteria as previously described (96, 97). For 4HA-tagged MavE constructs, following fixation in 10% neutral buffered formalin (NBF), the plasma membranes of infected hMDMs were differentially permeabilized using digitonin at 1 mg/ml in KHM buffer and incubated for exactly 1 min at room temperature (RT), and all wells were then immediately washed three times with 0.5 ml of KHM buffer (103). For all other confocal experiments, cells were fixed in −20°C methanol for 5 min and rinsed three times in 10% phosphate-buffered saline (PBS). For antibody labeling for MavE, mouse anti-L. pneumophila was used at a dilution of 1:500 and detected by Alexa Fluor 488-conjugated donkey anti-mouse IgG (1:1,000) (Invitrogen, Carlsbad, CA), and rabbit anti-MavE (ThermoFisher Scientific) was used at a dilution of 1:500 and detected by Alexa Fluor 555-conjugated donkey anti-rabbit IgG (1:1,000) (Invitrogen, Carlsbad, CA). For antibody labeling for localization, rabbit anti-L. pneumophila was used at a dilution of 1:750 and detected by Alexa Fluor 555-conjugated donkey anti-mouse IgG (1:1,000) (Invitrogen, Carlsbad, CA), mouse monoclonal anti-Cathepsin D (Abcam) was used at a dilution of 1:200 and detected by Alexa Fluor 488-conjugated donkey anti-mouse IgG (Invitrogen, Carlsbad, CA), mouse monoclonal anti-KDEL (Enzo) was used at a dilution of 1:200 and detected by Alexa Fluor 488-conjugated donkey anti-mouse IgG (Invitrogen, Carlsbad, CA) at a 1:1,000 dilution, mouse monoclonal anti-Lamp 1 (abcam) was used at a dilution of 1:200 and detected by Alexa Fluor 488-conjugated donkey anti-mouse IgG (Invitrogen, Carlsbad, CA) at a dilution of 1:1,000. For detection of 3×-FLAG-tagged proteins during transfection experiments, mouse monoclonal anti-FLAG (Sigma) antibodies were used followed by detection with Alexa Fluor 555-conjugated donkey anti-mouse (Invitrogen, Carlsbad, CA) at a 1:1,000 dilution. 4′,6′-Diamidino-2-phenylindole (DAPI) was used for all experiments at a 1:5,000 dilution. An Olympus FV1000 laser scanning confocal microscope was used in-house to examine cells as we described previously (104). On average, 10 to 20 0.5-μm serial Z sections of each image were captured and stored for further analyses, using Adobe Photoshop CS3. A total of 100 cells for each replicate were analyzed for the presence or absence of localization.

### Cloning of recombinant MavE.

The MavE (lpg2344) gene was amplified from L. pneumophila (Philadelphia) genomic DNA by PCR. Residues 183 to 204 are predicted to comprise a transmembrane (TM) region (Phobius program) (105). To clone only the soluble domain of MavE, we amplified the DNA sequence encoding residues 2 to 172. This construct terminates just after the final hydrophilic helical stretch and excludes the following loop and TM region. The MavE(2-172) insert DNA sequence was placed into pMCSG7 and pRL652 vectors by ligation-independent cloning (LIC), incorporating an N-terminal tobacco etch virus (TEV)-cleavable His6 or glutathione *S*-transferase (GST) tag, respectively (106, 107). His6-MavE (2-172) expressed poorly in BL21(DE3) pLysS (Promega) and GST-MavE (2-172) did not readily bind the glutathione resin. Running PsiBLAST on MavE showed that most homologous proteins have start sites corresponding to residue M38. To explore the possibility of a misannotated start site, we amplified MavE (39-172) using the following primers: sense, 5′-TACTTCCAATCCAATgccACTAGATTTGAAAGAAATTTCCTGATTAATAGC-3′, and antisense, 5′-TTATCCACTTCCAATgTTATTCGTCTTTGAGTTTGGCAATTAATTCTT-3′. As previously described, the MavE (39-172) DNA insert was placed into pMCSG7 and pRL652 vectors via LIC using the extensions underlined above. This construct of MavE was used for expression, purification, and crystallization trials.

### Protein expression and purification.

His6-MavE (39-172) was transformed into chemically competent BL21(DE3) pLysS cells and plated on LB agar containing ampicillin (100 μg/ml). A single transformant was inoculated into 20 ml of LB supplemented with ampicillin (100 μg/ml) and glucose (0.4%) and grown overnight at 37°C. This overnight culture was subcultured into 1 liter of terrific broth (TB) supplemented with ampicillin (100 μg/ml) and grown at 37°C. Once the cell culture reached an optical density (*A*_600_) of ∼1.0, the temperature was reduced to 18°C, 1 mM isopropyl-β-d-thiogalactopyranoside (IPTG) was added to the culture to induce protein expression, and the cells were incubated for approximately 16 more hours. Cells were pelleted at 6,900 × *g* for 15 min in a Beckman JLA 8.1000 rotor and stored at −80°C until further processed. Approximately 10 g of pellet was obtained from 1 liter of culture. Cells were resuspended in 30 ml of a lysis buffer (50 mM Tris [pH 8.0], 10% [vol/vol] glycerol, 0.1% [vol/vol] Triton X-100) and lysed two times at 35 kPsi in a cell disruptor (Constant Cell Disruption Systems, Kennesaw, GA). The lysate was spun at 21,000 × *g* for 30 min in a Beckman JA25.50 rotor. Supernatant was added to 5 ml of Qiagen NiNTA beads preequilibrated with 3 column volumes of a standard buffer (20 mM Tris [pH 8.0], 50 mM NaCl), and the beads were washed with 50 ml of standard buffer. Protein was eluted with standard buffer supplemented with 100 mM imidazole. Purified protein was concentrated to 18 mg/ml in a 10-kDa molecular weight cutoff Millipore centrifugal filter at 4,000 × *g*. The hexahistidine tag was cleaved by adding 100 μl TEV protease to 500 μl concentrated MavE and incubating overnight at room temperature. Untagged MavE was then loaded onto a Bio-Rad SEC70 or GE SEC75 column for buffer exchange and further purification. Peak fractions were collected and concentrated to 25 mg/ml for crystallization.

A seleno-methionine derivative of MavE (38-172) was produced by inhibiting methionine biosynthesis immediately prior to induction. Specifically, 100 mg lysine, phenylalanine, and threonine and 50 mg isoleucine, leucine, and valine were added to 1 liter of culture 15 min prior to induction. l-Seleno-methionine (60 mg) was also added to the culture, such that this version of methionine would be incorporated into overexpressed MavE during induction.

### Crystallization.

Both His6-MavE (39-172) and MavE (39-172) were screened for crystallization using Crystal Screen HT, Index (Hampton Research, Aliso Viejo, CA), JCSG Core II and Classics Suite (Qiagen, Toronto, Canada). His6-MavE (39-172) did not crystallize under any of the conditions tested, whereas MavE (39-172) produced crystals under several conditions. After optimization by the hanging drop vapor diffusion method, the best crystals were obtained at 20°C in drops containing 1 μl protein in 15 mM Tris-HCl (pH 8.0) and 50 mM NaCl mixed with 1 μl of reservoir solution (10% PEG 20000, 0.1 M citrate [pH 3.0]) and suspended over 500 μl reservoir solution.

### Data collection and structure solution.

The protein crystals were cryo-protected by transferring to 1 μl mother liquor containing 20% (vol/vol) ethylene glycol. Diffraction data were collected to 1.8 Å at the Canadian Macromolecular Crystallography Facility (CMCF) 08ID beamline, Canadian Light Source, using a MAR300CCD detector (108). Integration and scaling were carried out using the XDS software package (109) (autoprocess). MavE (39-172) harbors only one methionine at residue 51, and substitution of this residue for seleno-methionine produced sufficient anomalous signal to solve the structure by single anomalous dispersion (SAD) using the phenix.autosolve script. Refinement of the structure was carried out using phenix.refine (110).

### Statistical analysis.

All experiments were performed with at least three independent biological repeats, and the data shown are representative of one experiment. To analyze for statistically significant differences between three sets of data, the two-tailed Student’s *t* test was used, and the *P* value was obtained (* indicates *P* ≤ 0.05, ** indicates *P* ≤ 0.01, and *** indicates *P* ≤ 0.001).
