# Supplementary figures and images for: An Indispensable Role for the MavE Effector of Legionella pneumophila in Lysosomal Evasion
Source: mBio. 2021 Feb 9;12(1):e03458-20. doi: 10.1128/mBio.03458-20 (PMC7885109; doi:10.1128/mBio.03458-20)

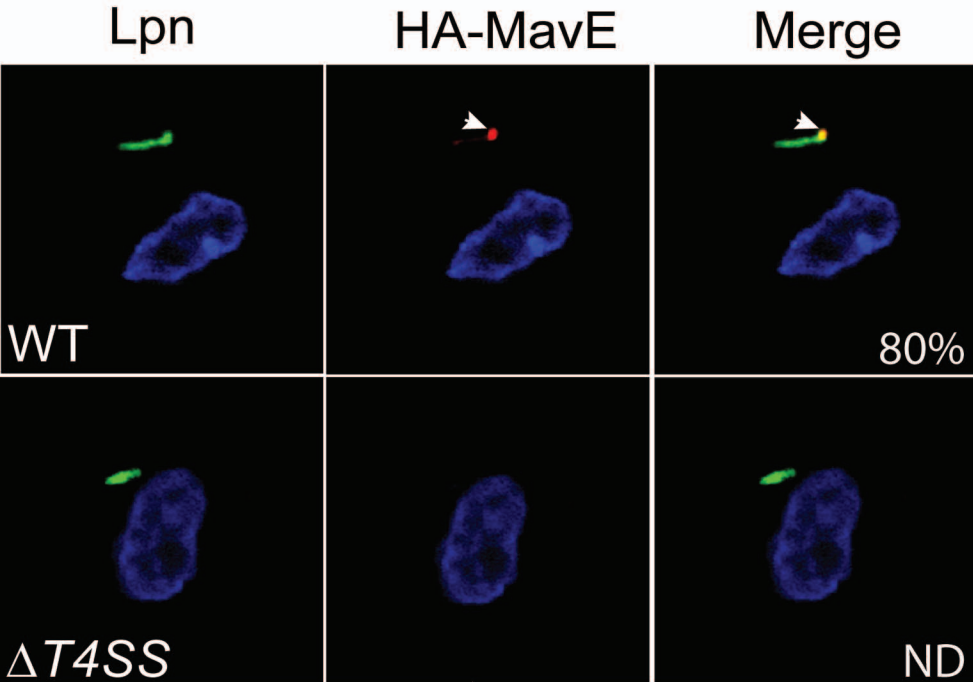

Supplemental Figure 1

Supplement: FIG S1 [file mBio.03458-20-sf001.pdf]

A

*in vitro* growth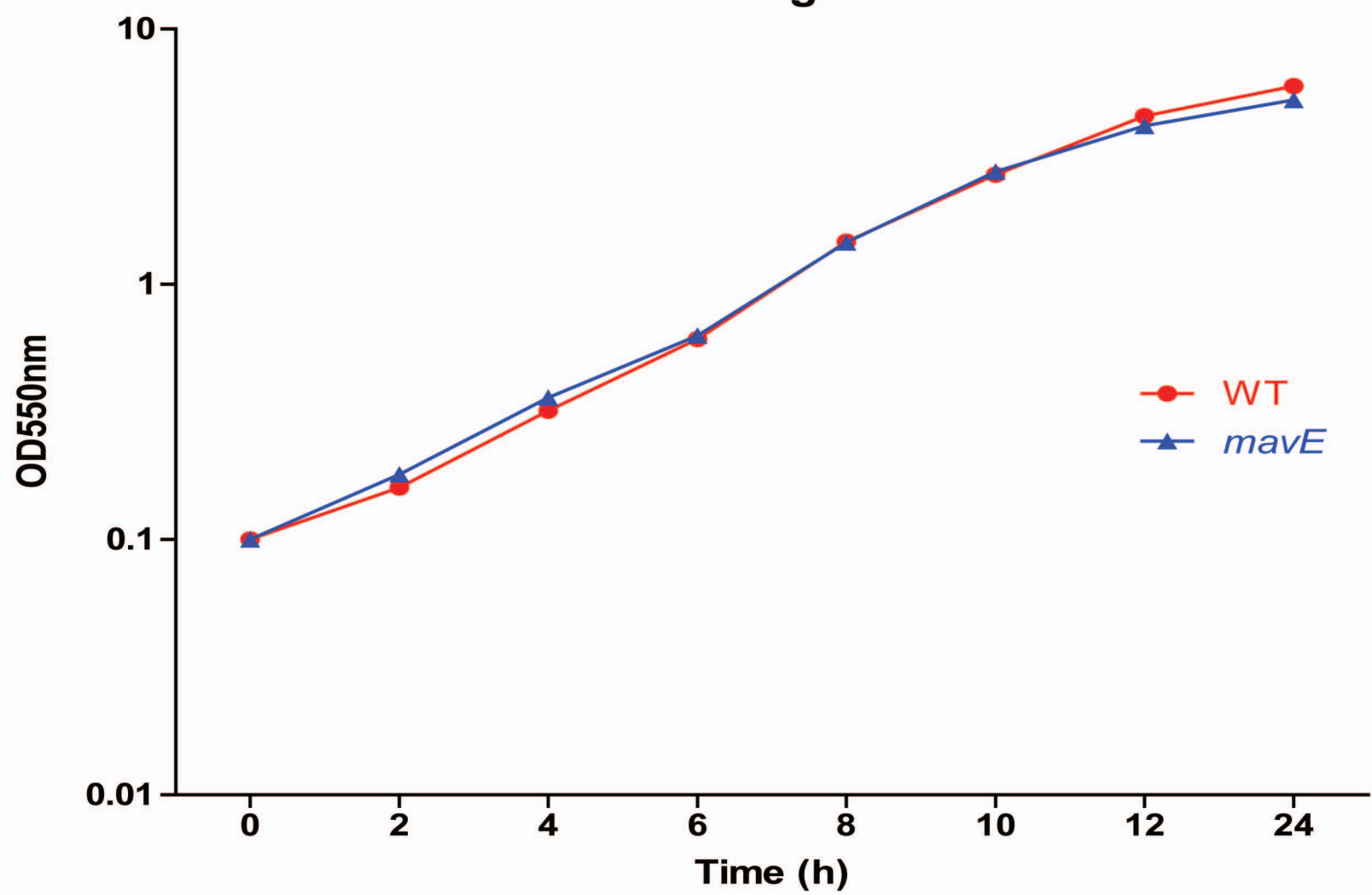

B

*in vitro* growth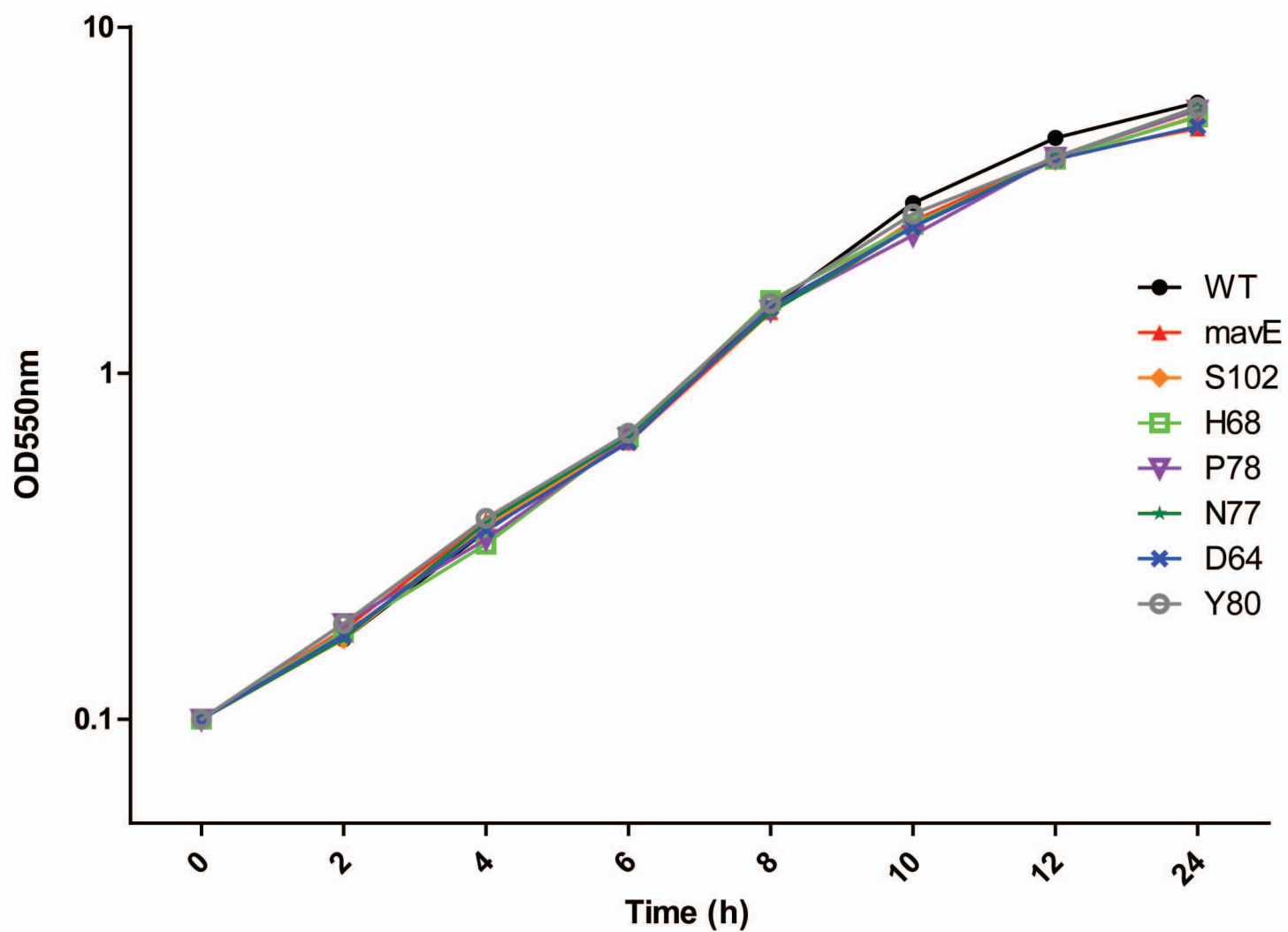

C

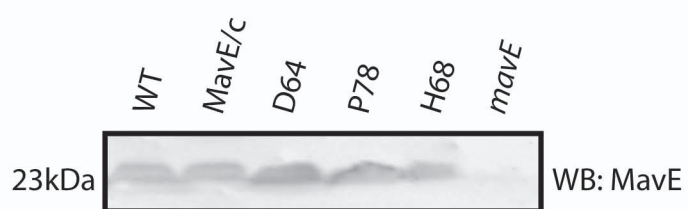

Supplement: FIG S2 [file mBio.03458-20-sf002.pdf]

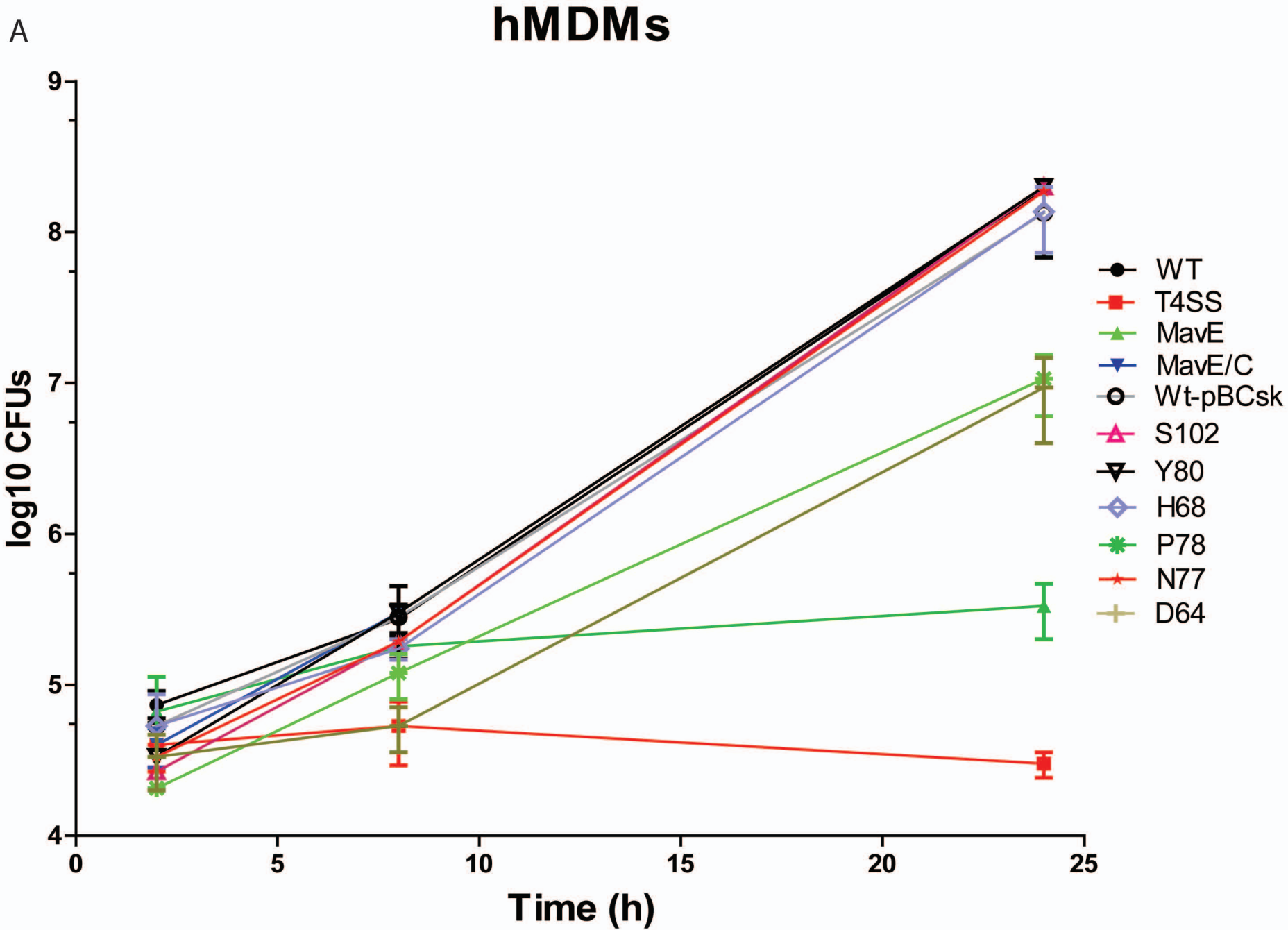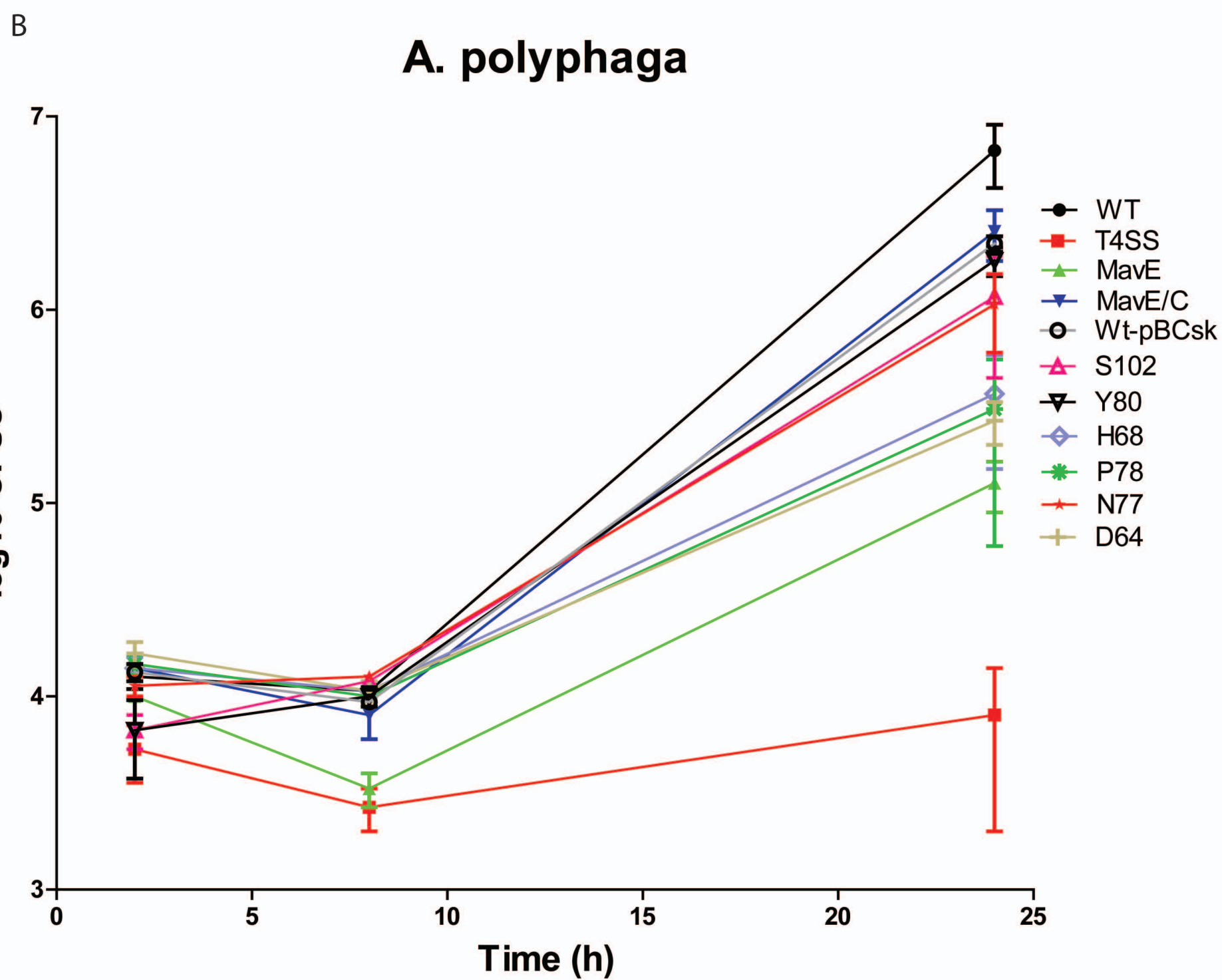

Supplement: FIG S3 [file mBio.03458-20-sf003.pdf]
